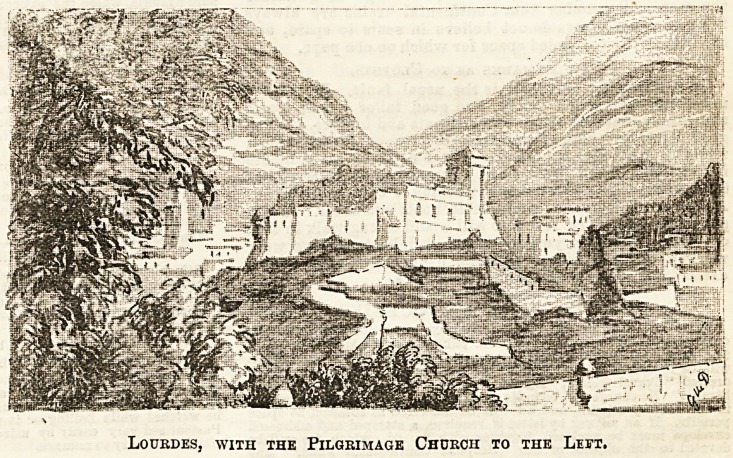# "The Hospital" Nursing Mirror

**Published:** 1899-03-18

**Authors:** 


### "The Hospital" Nursing Mirror.

Being the Nursing Section of "The Hospital."

[Contributions for this Section of "The Hospital" should be addressed to the Editor, The Hospital, 28 & 29, Southampton Street, Strand, London, W.C., and should have the word "Nursing" plainly written in left-hand top corner of the envelope.]

### News from the Nursing World.

### THE QUEEN'S HOLIDAY.

The Queen's trip to France has now become an annual event. It is in the highest degree desirable, from a medical point of view, that she should be spared the cold, trying winds of spring, and be enabled to rejoice in the sunshine and outdoor exercise upon which her health so greatly depends. Her Majesty left Windsor on Saturday, and arrived at Nice on Sunday afternoon. The journey occupied twenty hours. The French people accorded her the heartiest welcome, and her private apartments were filled with beautiful flowers. During the halt at Toulon the Queen expressed her sympathy with the sufferers in the recent explosion, and her intention of sending a subscription to the relief fund. Her Majesty's proverbial good fortune as regards weather did not desert her on this occasion, and she enjoyed a calm, sunny voyage across the Channel.

### A FORTHCOMING "AT HOME."

The tide of fortune seems to be setting favourably to the Royal British Nurses' Association. Not only has a refreshing if somewhat commonplace calm settled upon the meetings of the society, but its efforts to augment the benevolent side of its work are attaining a considerable degree of success. In May an "At Home" on a large scale will be given at the Hotel Cecil in aid of its funds, under the patronage of the Princess Christian, who is the president of the association. A limited number of tickets will be issued at one guinea each, and intending purchasers must secure them beforehand, as none will be sold at the door. Rumours as to the excellence of the dramatic and musical programme are afloat, and it is expected that some of the most celebrated actors and musicians will take part in it—Sir Henry Irving, Miss Ellen Terry, Lady Bancroft, and Mr. and Mrs. Beerbohm Tree amongst the number.

### NEW RECREATION GROUND FOR GUY'S NURSES.

Guy's Hospital nurses will be the envy of all their fellows when they are fairly in possession of the delightful new recreation grounds at Honor Oak, of which the purchase has lately been completed. Exercise and recreation are made great points of at Guy's, for it is Miss Nott Bower's creed that these are essential if her staff are to keep healthful and fit for their work. The scheme whereby the nurses have become possessed of their new ground has been carefully worked out amongst themselves, with the encouragement and help of the hospital authorities. All the nursing staff will, as a matter of course, be entitled to the privileges and advantages of the Recreation Club, and will pay a small yearly subscription, beginning at 5s. for first year probationers, and rising to one guinea for sisters, lady pupils, and extra members. For initial expenses the governors have lent the required sum at interest, and a piece of freehold land has been purchased outright at Honor Oak for the "perpetual enjoyment" of the nurses. Tennis courts are being laid out; there will be plenty of space for all sorts of games, and a cottage, where bicycles may be stored. The cottage, too, is to be cosily fitted up, so that when a "month's-end" two days' change of air comes round, nurses may adjourn there in twos and threes and picnic to their heart's content. It is hoped that by the summer this pleasant resort will be ready for use; it is only a short distance from London Bridge, and as by the revised rules the nurses have one whole and two half-days in a month, and three hours off on alternate days, it is sure to be in great demand.

### TRAINING NURSES IN SYDNEY.

We have received an account of the annual examinations, held in January, in Prince Alfred Hospital Training School for Nurses, Sydney, N.S.W. The papers were searching, and included one on cookery. Thirty-nine probationers presented themselves for examination, and many of the answers were very good. Only one probationer and two nurses failed to satisfy the examiners.

### DISTRICT NURSING IN SOUTH LONDON.

The annual meeting of the South London District Nursing Association was held, by the kindness of Sir Edward and Lady Durning-Lawrence, at 13, Carlton House Terrace on the 3rd inst. The nurses' home is at Battersea, and the staff consists of seven nurses and a superintendent. The income for the past year amounted to £1,330, including a sum of £378 realised by the concert at Stafford House last June; the expenditure was £880. Sir Edward Durning-Lawrence took the chair, and amongst those who warmly advocated the cause of the sick poor were Bishop Barry, Sir William Broadbent, and Mrs. Garrett Anderson.

### AN EPILEPTICS HOME.

Great strides are being made in the rational treatment for the epileptic. Undoubtedly, in the future, all children afflicted in this manner will be established in some country home, where, with careful training and in the enjoyment of agricultural work and rural pastimes, they will pass a guarded, innocent, and happy existence. All honour be to the pioneers of the movement which removes these unfortunate ones from their own homes, where they are a source of danger and misery to themselves and others, and secures them a haven of refuge from the storms and stress of a tempestuous world. To Lady Meath is due the credit of founding the Meath Home of Comfort at Westbrook, Godalming, for the benefit of epileptic women and girls between the ages of two and twenty-five. They are taught all sorts of handicrafts—needlework, basket-making, &c. Amongst the number of the purchasers of their wares are the Princess of Wales and the Duchess of Albany. Their taste for music is cultivated, and some of them learn to sing very nicely, whilst the preparation for their cantatas keep them happily occupied for months together. The special appeal issued for help last year met with a generous response, and the reserve fund has been restored to its original sum. A deputation of three of the members of the Bradford Board of Guardians inspected the home during the year, and inquired closely into its organisation. Every facility was afforded them in their quest for knowledge. The home is practically self-supporting. The members of the Girls' Friendly Society have secured a limited number of beds for their own society, for which the occupant pays a reduced charge of 10s. 6d. weekly. Ladies pay from one to two guineas, ordinary cases 12s. 6d., children 8s. weekly. The lady superintendent resident at the home will supply all information, both as to the privileges of subscribers and as to steps necessary to secure the admission of patients.

### SIR R. TEMPLE AT WORCESTER.

Sir R. Temple presided at the annual meeting of the Worcester City and County Institution for Trained Nurses, held early this month at the Guildhall, Worcester. In moving the adoption of the report he pointed out that the finances of the institution were satisfactory, but that in order to remain so the subscriptions must be well maintained. After a few remarks upon the working of the association he proceeded to make a comparison between nursing abroad and in England, from which the following salient points are taken: He thought that in our modern material civilisation there was nothing so important as hospitals, and that had been recognised by all modern nations—ever since the establishment of Christianity. If we English people were to fall behindhand in this respect our reputation would be very much lower in the eyes of the civilised world. He had seen hospitals in Italy, Austria, France, Germany, and Russia, and even in Spain, but the first two-named countries were most distinguished for the elaborate character of these institutions. Hospitals not only cured illnesses, but were the great centres of medical science. If we had not got them science would not be what it was, and that was a great reason for supporting them. If hospitals were to be of great benefit to modern society, they must have nurses, as any doctor would say that unless he had good nurses he could not cure his patients. If, however, they were to get women of the necessary physical power, nerve, and presence of mind, they must be paid. The improvement made in our hospitals within the memory of many present was something marvellous; and in no department of our national life had such improvement been so much needed.

### A HARDSHIP TO THE GUARDIANS.

The Irish Press is hotly debating the Local Government Board's (for Ireland) definition of a "trained" nurse. That it is the opinion of those who are, and have been, engaged in training nurses that a probationer learns little the first year, learns how ignorant she is the second, and really learns some nursing during the third, ought to vindicate the decision of the central authority. Nevertheless, everyone acknowledges that reforms undoubtedly sometimes occasion hardships to those who have bravely and adequately done their duty previous to the inauguration of a new state of affairs. There no doubt exist at the present moment many women whom the lack of the necessary three-year certificate debars from preferment, and yet whose native talent and wide experience, in spite of a shorter training than that now prescribed, have made them into first-rate nurses. And with such we sincerely sympathise. But the controversy appears in a sordid and not a generous light when upon closer examination it is seen that the undoubted hardship of these nurses is merely used as a shield to mask an attack upon the public purse. The return of half the trained nurses' salary to the board of guardians is a prize worth striving for, but it is a prize only to be given when the nurse is really trained. In the case in question it is the guardians who would profit, not the trained nurses. If the sympathy of these gentlemen is so keen with their uncertificated nurses, it is open to them to send them to a recognised training school for nurses for such a period as shall satisfy the central authority. Otherwise, excepting that they are not eligible for promotion to the highest post, the position of the nurses and their pay is what it was before the order.

### SHORT ITEMS.

Six nurses are wanted at once for work in Africa by the Universities' Mission to Central Africa, 9, Dartmouth-street, Westminster, S.W.—The Reading Board of Guardians are in receipt of a communication from the Local Government Board, accepting their certificates as qualifying the nurses trained at their infirmary under the present satisfactory arrangements for the post of superintendent nurse.—Swansea Hospital must now be added to the list of institutions requiring premiums with probationers. At a meeting of the Board on the 9th inst. it was resolved, in spite of some hot opposition, to require a premium of £10 from each accepted candidate.—Blackburn is at present considering the proposal to build a new nurses' home in connection with the District Nursing Association. A public meeting will shortly be held to further the project, at which the Mayor will take the chair. Amongst the contributions already received is one from Mr. Yerburgh for £100 towards the purchase of the site.—Mrs. Keeley, the oldest actress, died of influenza and pneumonia on the 12th inst. She was in her 94th year, and retained her personal charm until the last.—Several contributions have been received towards the establishment of a cottage home at Shoal Lake, Manitoba, in connection with the Victorian Order of Nurses.—The "battle of the sites" with regard to the new nursing home at Lichfield has at last been definitely ended. It has been decided to secure a good house in Sandford Street, which there will be no difficulty in selling in the event of a hospital being required.—An interesting series of lectures has been given recently on behalf of the Women's Industrial Council, at 39, Gloucester Square, on Wednesday mornings. Last week Miss Honnor Morten gave an excellent address on "Educational Ideals"; this week the subject was "Juvenile Offenders," and the lecturer the Rev. W. D. Morrison, D.D., formerly Chaplain of Wandsworth Prison. Next Wednesday Mr. J. R. Macdonald will speak on "Women's Work and Wages."

### Hints on the Home Nursing of Sick Children.

By J. D. E. Mortimer, M.B., F.R.C.S., formerly Surgical Registrar, &c., at the Hospital for Sick Children, Great Ormond Street.

(Continued from page 243.)

### MENINGITIS (ACUTE)—INFANTILE PARALYSIS—SURGICAL DRESSINGS.

### Meningitis (Acute).

Just as a slight jar of the bedstead may evoke severe pain in an inflamed joint, so in the early stages of this disease any disturbance of the brain and special senses, such as a bright light, a slammed door, a sudden question, may cause acute suffering. Hence, rest in the widest sense of the term is essential. A quiet room must be chosen, kept of moderate temperature and well ventilated. The patient should not face a light, either natural or artificial. Outside noises should be mitigated as far as possible, and conversation and the presence of visitors kept to the lowest practicable limit. When the child has to be taken up this should be done slowly, the head being carefully supported, so that an increase of pain and dizziness may be avoided. If cold applications are ordered for the head they must be kept continuously applied—the weight of an icebag should, of course, be taken off by slinging it or attaching it to the pillow. There should be no restraint of the child's movements, and if the bedclothes cannot be kept in place by fastening them with tapes and clips to the bedstead, it is better to dispense with them altogether and clothe the child in a long flannel nightgown fastened below the feet, or in a sleeping suit. It may be necessary to arrange pillows or cushions so that there is no fear of the child rolling off the bedstead or striking the wall in a convulsive fit. The usual precautions must be taken in regard to the evacuations and for prevention of bedsores. In this disease (and when delirium occurs in other conditions) the nurse must see that there is no remediable cause for increased restlessness, just as she would in the case of a crying baby. Incessant, half-conscious talking may be best met by occasionally saying a few words in a calm, reassuring tone, neither keeping entirely silent nor encouraging the over-active brain by too-frequent reply. The patient should not be told to try and force herself to keep quiet; any repression of this kind, like forcible restraint of a convulsed limb, is apt to make matters worse.

In mere chronic diseases of the brain and its membranes, such as tumours, hydrocephalus (water on the brain), &c., a similar plan of treatment should be adopted so far as may be necessary. The nurse should take particular note of any convulsive twitchings, rolling of the head, or boring it into the pillow, increased drowsiness, and so forth.

### Infantile Paralysis.

Although infants and young children are liable to many forms of paralysis, there is a certain disease to which alone the term of "infantile paralysis" is strictly applied. It begins suddenly with a more or less severe feverish attack, lasting from a few hours to a few days. As this passes off there follows a stage of general weakness, the child is limp and tender, disliking to be touched, and may pass evacuations into the bed. It is then found that certain movements cannot be performed from the nerve supply of some of the muscles having been damaged. These soon become flabby and wasted and the limb cold and bluish.

The object of treatment is now to improve the nutrition of the limb, especially of the damaged muscles and nervous structures with which they are so intimately connected, and to promote the growth of tissues which may replace such as have been hopelessly destroyed. Patience and perseverance are most essential to success. It may be necessary to continue steady and systematic treatment for months, or even years, before it can be said that further improvement is impossible. The affected limb must be kept warm both day and night by long woollen stockings, flannel knickers, or, if necessary, a long bag lined with wool and quilted. There must be no garter, strap, or anything similar constricting it; friction and warm covering should be relied on rather than hot-water bottles, &c.; if these are used they must be well wrapped up, as the skin is very apt to blister. If any apparatus is used care must be taken that the limb is not encircled and its circulation interfered with, and that there is no pressure which may cause a sore. The limb should be daily douched with salt and water, first hot then cold, and rubbed dry and warm afterwards.

Massage should be done for a quarter of an hour twice a day. Some simple oil should be used, and the work always done with the palm and ball of the thumb from the fingers or toes towards the trunk, not in the contrary direction. The object is not so much to apply friction to the surface of the skin as to knead and squeeze the muscles.

Supposing one leg affected, take it in the left hand and (1) rub from foot to loin, front and back, with firm, slow strokes, avoiding any part, such as the shin, where the bone is thinly covered; then (2) grasping it above the ankle with both hands, squeeze the flesh so that as you work upwards the calf, and then the thigh, is flattened out between the thumbs in front and the fingers behind; then (3) rub round from ankle to groin with both hands in opposite directions as if wringing sheets; next (4) flip the limb all over until some redness is produced; and finally (5) gently rub up and down all over the surface.

Passive movements, such as bending the thigh quite up on the abdomen, are useful, and "resisted" movements are very important; for instance, whilst the nurse steadies the knee with her left hand she may slowly push the foot up with her right and try and get the child to push down against her. The doctor will no doubt devise a system of exercises of this kind and also of active movements, such as tricycling, pulling at a weight, &c., according to the muscles affected. Light go-carts with broad supports and other appliances are useful, but the child should as a rule be encouraged to do without them as much as possible. A watch must be kept for stiffness or deformity due to unopposed action of the sound muscles, and here, as in congenital contractions, much may be done by daily manipulation.

### Surgical Dressings.

The skin of a child is more liable than that of an adult to be irritated by certain antiseptics, and under exceptional circumstances these may be absorbed into the system. Carbolic acid in such cases induces vomiting, stupor, and the urine is tinged greenish-black, and there may be even coma and collapse. Mercuric (alembroth, cyanide, sublimate) dressings, ointments, &c., may give rise to colic and diarrhœa, and iodoform to loss of appetite and depression, pyrexia, delirium, alternating with drowsiness, and other symptoms resembling those of septicæmia or meningitis. When a dressing has to be changed, or other surgical work done, everything that may be wanted should be put ready, so that there may be no delay when once begun. The preparations should be made outside the room, or at least out of the child's sight. Whilst doing all that is possible to avoid pain (*e.g.*, by thoroughly soaking a dressing that has stuck), the task should not be prolonged to the child's disadvantage by spending much time in arguments and entreaties.

### Antiseptics for Nurses.

BY A MEDICAL WOMAN.

### XXV.—STEAM DISINFECTING APPARATUS.

*Continued.*

THE Equifex low-pressure stove is another of the cheaper and simpler forms of apparatus, and is intended for use with steam at a pressure of 10 lbs. for every square inch. It is also made for use with steam at 3 lbs. pressure (but this is not recommended), as well as for current steam at atmospheric pressure. As a rule, a temperature to produce a pressure of 10 lbs. requires a stove to be made with sufficient margin of strength to provide for the accidental failure of the safety valve, and, of course, the cost is in proportion. To provide a stove in which such margin is reduced to the limits necessary for the production of a cheap apparatus, the Equifex is made without any safety-valve at all, and the pressure is regulated by the height of a water-seal contained in the cylinder adjoining the stove. In this way the steam, as its pressure increases, depresses the column of water in its outlet tube, dipping into the water-seal, and, when the desired pressure is obtained, the bottom of this tube is exposed, and the steam has a free way to escape into the open air or flue-pipe.

SATURATED-STEAM STOVE FOR ABSOLUTE DISINFECTION.

The pressure to which the stove is usually made is of 2 lbs. to the square inch, corresponding to a height of 4½ ft. in the water-seal, but this pressure can be increased where desired at a small extra cost. The working of the apparatus is as follows: Water is filled into the boiler, which is placed immediately below the disinfection chamber, and, in the case of the larger stoves, is made in two cylinders connected by a pipe and having a grate running across the bottom of both. The water is fed in through a ball valve in an adjoining vessel, and the cistern is placed at a height exceeding the maximum pressure of the steam. As an alternative, it can be fed by hand between each time of using. The steam, when formed, passes through a short pipe connecting the disinfection chamber with the boiler, and round a screen which extends round the inside of the stove, emerging at the top of the chamber. The object of this coil of pipes is to impart to the steam as much heat as it has lost by radiation through the walls of the disinfection chamber, and at the same time to prevent undue condensation, as by this passage the steam will have been freed from all superfluous moisture, which flows back into the boiler. In this way the need for a steam jacket is obviated. The steam next traverses the things to be disinfected, and escapes at the bottom end of the apparatus through a pipe which leads it on to the water-seal. The disinfection proper begins when the steam first comes out of the water-seal, and is accordingly carried out by means of current steam under pressure which the water seal determines. The process of disinfecting is carried out as follows: The clothes having been introduced and the door closed, steam is blown into the apparatus at the top and allowed to pass out at the bottom by another pipe, thus removing the greater part of the air in the chamber and from the clothes themselves. After this has gone on for three to four minutes the escape pipe is closed, and steam is admitted to the chamber up to a pressure of 10 lbs., and is maintained for five minutes at this pressure; it is then allowed to escape and fresh steam is again admitted. The pressure is intermitted once more, and the steam blown off at the end of a second five minutes. The object of these intermissions is to completely remove the air from the pores of the articles to be disinfected by the sudden expansion of the film of water previously condensed on their surface. The time necessary for disinfection depends on the class of objects to which it is applied, and would ordinarily be half an hour. When the disinfection is over thin objects are taken out and shaken and require no further drying. To dry thick articles air is introduced at the bottom of the chamber from tubes round which steam has circulated, and in this way the drying is effected very rapidly and without any risk of injuring the articles by scorching or otherwise. The apparatus is fitted preferably with two doors, which are secured by the Equifex safety lock-nuts, and the whole apparatus is complete in itself and can be worked by any labourer who has once been shown how to do it. In this disinfector the steam used is under pressure, but instead of removing one-half to two-thirds of the air by forming a vacuum the steam is allowed to escape freely for a few minutes before the disinfection begins. Advantage is also taken of the steam being saturated, and the consequent direct correspondence between the pressure and its temperature, to allow the steam to work a recording pressure gauge, of which the readings indicate precisely the steam temperatures obtained in every operation, and thus serve as a check on the operator. The advantages claimed for the Equifex are: (1) It uses saturated steam only, the temperature above boiling point being obtained without the use of superheat, either through jackets or saline solutions; (2) the pressure is controlled without any mechanical moving parts, and could not possibly give rise to an accident; (3) the disinfection is carried out by means of current steam, which gives the most reliable means of eliminating the air; (4) the apparatus can be fitted with a recording gauge; (5) the whole apparatus is self-contained, and neither requires skill to work it nor entails liability to accident when improperly worked.

Thursfield's disinfector is a favourite stove on the Continent. Originally it was constructed for disinfection by the combined use of hot air and steam. The steam was generated in a separate open boiler, having a steam outlet connected in such a way with an apparatus for heating air or with the chimney as to cause either hot air alone or mixed with the products of combustion to be drawn into the chamber together with the steam. The modern types of this machine are horizontal, circular in section, and are provided with a steam jacket, the latter being partly filled with water and acting as a boiler, and the furnace being underneath. As the boiler is open steam can only be generated at atmospheric pressure, and is used on the current system. Condensation is minimised by the steam jacket, but there is no provision made for drying the clothes after disinfection. This apparatus can be obtained made either for moving from place to place or for stationary use.

Another disinfector largely used abroad is Schimmel's, which in many ways resembles the Equifex as regards general arrangement. It is either circular or oval in section, and is fitted with one or two doors as preferred. Low-pressure steam is used, and is obtained from a separate boiler. The chamber has no steam jacket, but in the lower part of it a double coil of pipes is placed which are heated by steam. The articles to be disinfected are disposed in a suitable carriage and placed in the chamber, which has been previously warmed for about half an hour, and the doors securely closed, and the clothes remain for another half hour so as to get thoroughly warmed before steam is admitted to the chamber for a period of half an hour, and then hot air is passed through for about a quarter of an hour, after which the clothes can be removed, but they are not perfectly dry. Disinfection by this apparatus is very effectual, but slow.

Mason's disinfector consists in a chamber built of brick, with a furnace beneath it which generates steam and also warms the air. Either moist or dry heat may be used, and either separately or in combination. A damper is provided to automatically regulate the heat. All air leaving the chamber is purified by passing through the furnace, which is constructed to burn the cheapest kinds of fuel. This stove has been reported on favourably, and is largely used in some parts of the country.

### Bed Sores.

There are three main points to be considered in the treatment of bed sores:—

(a) The sore is the result of pressure, therefore the pressure must be relieved. This can often be best done by means of a water-bed, by which the pressure of the weight of the body is distributed over a larger surface than is the case when a bed of a harder or less flexible character is in use. It must be remembered, however, that in very emaciated subjects a water-bed is not always a satisfactory arrangement. Water is heavy, and therefore a water-pillow must be strong. Hence it is not quite as flexible as could be wished, and does not very accurately mould itself to the form of the body, so that even the best water-pillow throws undue pressure upon the sacrum and the projecting portions of the iliac bones. Air-cushions are much more flexible, and those with a hole or a hollow in the centre are often of great service. In paralytic cases, however, it is not always so easy as it might seem to get these pillows into position, and it is often useful to have at hand much smaller cushions which can be easily pushed in at one side or the other of the part to be relieved even above a water bed or pillow. They can be made of many layers of old blanket piled one on the other, "mattressed" by a few through stitches, and covered with linen. They should be about the size and thickness of an A B C railway guide. Such cushions can be easily pushed in close up to, but not to touch the sore area, and while they give great relief to the pressure, their own points of pressure, being variable, need not do harm—which is more than can be said of some of the larger and firmer kinds of pillows which one sometimes sees in use. Frequent change of posture is one of the most valuable means of relieving pressure since it also at the same time ventilates the parts involved and improves the circulation.

(b) The sore is much more apt to form when the vitality is low; therefore, wherever bed sores threaten questions of nutrition have to be considered.

(c) The final act in the production of a bed sore is the access of septic influences; therefore, both as a means of prevention and of cure, cleanliness is all essential.

In regard to prevention, washing is the first and most important matter. This does much besides merely removing dirt. During the time that the back is being washed the pressure is entirely removed. It is desirable, therefore, that every pains should be taken to place the patient in as comfortable a position as possible while it is being done, so that the performance may take place in a somewhat leisurely manner.

Then although the actual washing, that is the removal of the perspiration and the dirt, should be done with warm water, the part should be alternately dabbed with hot and cold water several times during the process, so as to stimulate the circulation. After the washing is over and the parts are dry they should be gently rubbed with the soft hand for a time with the same object, and it is often advantageous to use starch and zinc powder or French chalk on the hand. The greatest care should be taken to get all the skin quite dry before turning the patient back again.

Many nurses use various applications to the back after washing. Most of these things, except for the spirit of wine that they generally contain, are useless. Spirit of wine (or brandy or whisky) is useful in two ways. First of all it is a good antiseptic, and then, if pretty freely dabbed on and then wiped off again, it takes with it a great deal of the water which the skin has absorbed, and so promotes a more complete drying than could otherwise be obtained. The idea that the action of spirit is to harden the skin has no doubt led to the use of alum and such like astringents, but it is open to much question whether such substitutes are of any service. When a crack is just appearing it is possible that a touch of collodion may check its extension, but there are many very obvious objections to covering large areas of skin with any impervious material, such as collodion or the common mixture of alum and white of egg, or even of white of egg and spirit. Perspiration is retained and a cracky condition is induced which is difficult to get rid of, and also difficult to clean. The skin should be kept healthy, and healthy skin is soft. Sometimes the use of the spirit of wine tends to make the skin a little scurfy, in which case, after it has been applied and the skin has dried, a very tiny touch of lanoline may be gently rubbed in.

When bed sores have once formed the surgeon in charge must decide on what is to be done. Cleanliness, however, is absolutely essential It must always be remembered that when a patient lies on a mackintosh any antiseptic that is used, even although no oiled silk may be applied to cover it, is practically locked in, and tends to become a hot dressing. Frequent changing them, then, must be the rule, and in view of the very feeble nutrition of the tissues it is probably best to use the stronger antiseptics, such as carbolic or sublimate lotion, merely for washing purposes, and always to dress the sore with such mild applications as boric acid lint or boric acid lotion, or the old-fashioned resin ointment. If stimulating ointments are used, it is important to prevent them from running over the sound skin. This is probably best done by covering the surrounding skin with a piece of lint spread with an innocuous ointment, such as the benzoated zinc ointment, and having a hole cut in it corresponding with the sore, and then dressing the sore through the hole. But ointments are generally very unsatisfactory dressings for the back, and they are very destructive to mackintoshes. There are many dodges which help a sore to heal, such as occasionally puffing it with a little iodoform, or touching the edges with a stick of nitrate of silver, or painting it over with friars' balsam. Probably most of the older methods were in essence antiseptic, and much of the good which they did can be obtained by antiseptic washing, and frequent dressing with boric lint.

Where bed sores are definitely the result of neglect, that is of dirt and pressure, and where it is possible to alter the position so that the pressure can be relieved, perfectly brilliant results are sometimes obtainable by the surgeon scraping off the "proud flesh," and after cleansing the sore dressing it as he would an operation wound. This, however is a surgical affair.

### A Book and its Story.

### POISON ROMANCE AND POISON MYSTERIES.

MR. THOMPSON has given us in the book under notice* a work that will be read with much interest by all who take any interest in the science of toxicology. The book is intended for the non-scientific reader, but even professional men who have not made a special study of the subject will find much in it of interest.

Mr. Thompson commences his subject with the poisons of antiquity, and the chapter is a most interesting one. Under the heading of "Poisons and Superstition," the writer introduces us to mandrake, hellebore, and the Calabar bean. Round the mandrake, of course, a large amount of superstition has centred from the time of Jacob onwards. "Pliny says 'Mandrake is taken against serpents, and before cutting and puncture, lest they be felt. Sometimes the smell is sufficient.'" In Shakespeare's time mandrake still kept its place in public estimation as a narcotic. Thus we have Cleopatra asking for the drug that she may "sleep out this great gap of time while her Antony is away." "The Greeks believed that when the mandrake was dragged up from the earth it gave a dreadful shriek and struck the daring person dead who had the presumption to pull it up. The method of obtaining it, therefore, was by fastening the plant to the tail of a dog, who thus drew the root from the ground. The shriek was supposed to be due to an evil spirit who dwelt in the plant."

In the chapter on royal and historic poisoners we are presented with a long list of notabilities who habitually got rid of persons displeasing to them by poisoning. Best known of these is, of course, Lucretia Borgia, but Robert Dudley, Earl of Leicester, Prime Minister and favourite of Queen Elizabeth, seems to have been the most unscrupulous poisoner in all this gallery, and a long list of the crimes of which he was suspected is given. "He is said to have kept in his employ several needy but unscrupulous physicians, ready to administer the 'Italian comfortive,' as the poison was called, at his bidding."

Mr. Thompson's account of the professional poisoners is interesting. "In the early Christian era poisoning became quite a profession, and convenient individuals could be hired with little difficulty to administer a deadly dose to an enemy or rival." "In mediæval times a law was passed in Italy rendering the apothecary who knowingly sold poison for criminal purposes liable to a heavy penalty, and yet secret poisoning was practised to a very large extent, and there were probably many like the poor apothecary of Mantua in 'Romeo and Juliet,' who in response to Romeo's demand for poison replied, 'My poverty and not my will consents.' From the fifteenth to the seventeenth century two great criminal schools arose in Venice and Italy." In the sixteenth century we find John of Raguba, a Franciscan brother, offering the notorious Council of Ten his services as a poisoner for a pension of 1,500 ducats and stating a regular tariff for the removal of notabilities. Some of the recipes used at this time seem unnecessarily complicated: "Take of the powdered leaves of Aconitum lycoctonum, Taxus baccata, with powdered glass, caustic lime, sulphide of arsenic, and bitter almonds. Mix them with honey, and make into pills the size of a hazel nut." It is difficult to imagine anyone swallowing such a nauseous bolus unintentionally. Italy seem to have been the hotbed of poisoning in those days, and "it is a curious fact that most of the notorious poisoners in mediæval times were women, and, indeed, in later years the frail sex seem to have retained a special predilection for this form of crime." "In the seventeenth century the mania for poisoning seems to have spread to France," and there it flourished exceedingly. The author gives details of a few plots for wholesale poisoning, notably of one a century and a half ago, when an attempt to poison all the Christians in Malta was discovered and frustrated. Another plot in Lima was rather amusingly frustrated by a chemist selling a would be poisoner 200 lbs. of alum for a similar amount of corrosive sublimate.

From a general survey of poisons and poisoners of the past, Mr. Thompson proceeds to take the various commoner poisons in detail, a chapter descriptive of the drug being followed by an account of one or more famous cases in which the poison was the cause of death. Arsenic is the first poison thus treated, and as explanatory instances we are given the Lafarge, Madeline Smith, and Maybrick cases. Mr. Thompson does not mention how commonly arsenic is used in India for cattle poisoning, often for no other reason than a grudge against their owner; but probably in many cases cattle are poisoned by skinners simply for the sake of their hides. The great ease with which arsenic can be procured in India, of course, makes it a favourite agent. Many cases of poisoning of human beings occur in India which are never reported or heard of. Often poison is not suspected, and when it is suspected, so great is the fear of the police, their exactions, and their methods, that in most cases the victim's family are only too glad to hush the matter up. Apropos of arsenic Mr. Thompson tells several good stories which are worth reading.

Mr. Thompson mentions a curious idea about aconite. "It was used by the ancient Greeks and Romans to destroy life, and they believed they could cause death to take place at a certain time by regulating the dose of poison. Thus Theophrastus writes, 'The ordering of this poison was different according as it was designed to kill in two or three months or a year.'"

"At Marseilles a poison was kept by the public authorities, of which hemlock was an ingredient. A dose of this was allowed by the magistrates 'to anyone who could show a sufficient reason why he should deserve death.' Valerino Maximus observes, 'This custom came from Greece, particularly from the Island of Cios, where I saw an example of it in a woman of great quality, who having lived very happy ninety years, obtained leave to die this way, lest, by living longer, she should happen to see a change of her good fortune.'"

A curious note follows regarding suicide courts. "When the Greeks and Romans recognised the impossibility of suppressing suicide, they decided to establish tribunals, whose duty it should be to hear the applications of those persons who wished to die. If the applicant succeeded in showing what the tribunal considered good cause for quitting life, his prayer was granted and he destroyed himself under the authority of the court. In some instances the court not only sanctioned the suicide, but supplied the means of self-destruction in the shape of a decoction of aconite and hemlock. If anyone applied for permission to end his life and was refused, and, in defiance of the decision, committed suicide, his act was illegal. The Romans in such cases confiscated the property of the deceased; the Greeks held his memory as dishonoured, and treated his body with indignity." By the way, is *Bish* the distinctive Hindustani name for aconite? *Bish* in Hindi is used for poison generally, though possibly in the districts in which aconite is gathered, it may be locally used for aconite in particular.

With an amusing chapter on "Poisons in Fiction" Mr. Thompson closes his book. The excellent way in which the Scientific Press has turned out the volume is deserving of the highest praise.

* "Poison Romance and Poison Mysteries." By C. J. S. Thompson. (London: The Scientific Press, Limited. 1899. Price 6s.)

### The Cookery Exhibition at the Imperial Institute.

Of the many object lessons given to the public, the Universal Cookery and Food Exhibition, held at the Imperial Institute from March 7th to 11th, must surely be reckoned one of the most important. Nevertheless, it was well calculated to inaugurate a severe attack of dyspepsia, elaborate arrangements having been made by the various stallholders not only to afford visitors opportunities of tasting the many tempting wares, but to make it exceedingly difficult to avoid doing so.

The first things a cook (or a nurse with an interest in her patient's diet) thinks of are stoves and utensils. There was consequently a large display of these necessary appliances. Messrs. T. S. Knight and Sons (222, Great Portland Street, London, W.) showed their new and improved portable range named "The Portland;" the Gaslight and Coke Company (showrooms, 1 and 2, Grand Hotel Buildings, Charing Cross, W.C.) exhibited their clean-looking enamelled stoves of all sorts and sizes, some just the thing to fix up in a room adjoining that occupied by the patients, wherewith a nurse could concoct special delicacies. The fact that they can be hired adds to their usefulness. For a fixture the "Main" gas cooker (list from 109, Farringdon Road, E.C.) has many advantages, and is largely used in London. The Davis Gas Stove Company (Camberwell, S.E.) had their Record Automatic Boiling Stove on view. It is an invention which is of decided advantage to the economical, and the patent addition can be applied to any other gas stove.

Perhaps the next thing a nurse values after her cooking stove is an ice safe. For this Messrs. Jollie and Co. (121, Charterhouse Street) cater temptingly. The outside of their cabinets is very handsome, so to fit them for use in places where they can be seen. Messrs. Ebenezer Roberts and Son (Camberwell, S.E.) displayed their ice-cream freezer. The cheapness of this handy little machine will recommend it to nurses who are in attendance of cases requiring milk diet. Ice-cream is so often now ordered as a palatable change in prolonged milk feeding.

Earthenware utensils have always been used abroad, and with the improvement in English cookery they are becoming more appreciated by our own cooks. Messrs. J. G. Wadman (7, East Chapel Street, Mayfair, W.) had specimens of all sorts and kinds, from the homely brown pot which finds a place on the kitchen stove to the artistic green and gold enamelled dish designed for table use as well. The "Bella Wattee" Patent Teapot (244, Oxford Street, W.) has been described already in our columns, and a capital invention it is. Rowland's Patent Hygienic Pie-cup (Woodbridge, Suffolk) is a cheap and natty little contrivance for keeping up the pastry of pies from the gravy or juice. It is also handy to put into a saucepan of milk, as it prevents the liquid boiling over. The use of cheap convenient milk sterilisers (St. Matthew's Works, Ipswich) is sure to become more common now that the public are alive to their value. Aymard's Patent Milk Steriliser for household use is one of the simplest and best; it is very widely employed by Governmental departments, hospitals, and public schools. Mincing machines are invaluable assistance in the preparation of beef teas and invalid dainties. The Universal Food Chopper (agents, Peck and Co., 8, Bradford Avenue) can be regulated to chop numerous articles to different degrees of fineness, whilst anyone who has used the "Alexander" Mincer (42, Moor Lane, Fore Street) can vouch for its utility.

Passing from appliances to food preparations, the most novel is "Protene" (the Protene Company, 36, Welbeck Street, W.). It is, as one advertisement calls it, "milk in sacks." In appearance it resembles a heavy white flour, and is manufactured from certain parts of pure fresh milk. It may be used in various ways for making cakes, biscuits, &c. The "Manhu" Diabetic Foods (88, Charing Cross Road) are now well known, and are of great service to diabetic and gouty patients. "Bermaline" is an especially milled flour, from which only the outer husks of the grain have been removed. Moreover, a second portion of the inner husk, which contains the greater part of the wheat, is also added. Bermaline flour is supplied to bakers with directions for use. There was also on view yet another digestive bread named the Opmus (John Thompson and Son, Boston, Lincs.), whilst Quaker Oats was greatly to the fore.

Dried vegetables are becoming plentiful, and some very fine specimens were to be seen steeping beside the dried samples. The manner in which many of them resumed their original appearance was wonderful. The British Preserving Company's (Rayne, Essex) and the "Gye Process" (21, John Street, Adelphi, W.C.) vegetables appeared very good—the latter firm apply the process to meats also. "Ovo," or dessicated egg (the McCredy Manufacturing Syndicate, Limited, 248, Gresham House, Old Broad Street, E.C. is the invention of an Irishman, and Canada is now the centre of its preparation. Pancakes and egg flip and other egg dishes were successfully made with it under the eye of the beholder. Turtles and turtle food in bottles, in tins, in flakes, and alive were exhibited by Mr. John Lusty (8, Parnham Street, Limehouse, E.), and Messrs. Brand and Co. (11, Little Stanhope Street) had an excellent display of their numerous and proved invalid specialities, whilst Bovril and "Vejos," were prominent amongst meat juices. Refined suet and fats were represented by Hugon's "Atora" beef suet, Dickson's (Edinburgh) Scotch beef suet, and "Albene," a vegetable fat which has made a place for itself amongst cooks. The gelatines shown by Messrs. Cox's (Gorgie Mills, Edinburgh had a very good appearance. It is now one of those used in the National School of Cookery. Norfolk Cyder (97, Queen Victoria Street, E C.) and invalid stimulants (A. Streiff, 39, Eastcheap) were exhibited; and there were numerous interesting tea and coffee stalls. Efforts to secure a good essence are still made with more or less success. Three makers (Messrs. John Travers and Sons, 119, Cannon Street, E.; Messrs. Robert Jackson and Co., 172, Piccadilly, W.; and Messrs. Yeatman and Co., Denmark Street, E.) were represented in this line. Messrs. Peek, Frean, and Co., the Britannia Fruit Preserving Company, Messrs. Veerasawny (chutneys, &c.), Messrs. Shippam (Chichester), Messrs. Th. Marquis and Cie. (74, Tottenham Court Road, W.), Messrs. Streimer and Co. (Ward Road, Stratford), Messrs. Heinz (Pittsburgh), each and all presented a bewildering variety of confectionery and prepared fruits, foods, and condiments. All were not suited for invalids, but many were. The Western Counties Creameries (T. H. Beard, Yeovil) had an attractive display of butter.

The Nautical School of Cookery had various competitions, and the little bluejacket cooks were intent on their demonstration, turning their rations into appetising dishes very deftly.

The Technical Education grants of the various County Councils encourage these courses of instruction. For 2s. a dozen lessons are given to any *bona-fide* sailor. Mr. T. E. Adkins is thus employed by the London County Council at the Wells Street Sailors' Home, E.

The Army camp cooking demonstration created much interest, the earth ovens in full operation in the ground attracting many visitors.

### Everybody's Opinion.

[Correspondence on all subjects is invited, but we cannot in any way be responsible for the opinions expressed by our correspondents. No communication can be entertained if the name and address of the correspondent is not given, as a guarantee of good faith but not necessarily for publication, or unless one side of the paper only is written on.]

### PLAGUE NURSING.

Miss Margaret Campbell writes: Will you kindly correct a mistake which has appeared in the current issue of The Hospital "Nursing Mirror," under the heading, "News from India," where it is stated that "Miss Campbell did not remain over three months" (*i.e.*, in India on plague duty)? I was in India from February 12th, 1898, to August 30th, 1898, three months of which time I spent very hard at work in the Plague Hospital at Modykana in Bombay; and I left India not because I could not "make more money or have more comfort," but because it was impossible to live under the conditions imposed upon us by the Calcutta authorities.

### Appointments.

Huddersfield Infirmary.—On the 13th inst. Miss K. M. Mackenzie was appointed Matron of the above. She was trained at the Western Infirmary, Glasgow, and at the Royal Hospital for Sick Children, Edinburgh. For three and a half years Miss Mackenzie held the post of sister and assistant matron at the Jenny Lind Infirmary, Norwich, where she had entire charge in the absence of the matron. In 1893 she became night superintendent at the Bradford Royal Infirmary, and a year later matron of the Bradford Incorporated Nurses' Institution.

General Hospital, Barbadoes.—Miss Caroline Walker has been appointed Matron of this hospital. She was trained at the London Hospital, and was afterwards at the Victoria Hospital for Children, sister at the Government Hospital, Gibraltar, and the Government Civil Hospital, Hong Kong, and last held the appointment of matron at the Hospital of St. Cross, Rugby.

Hospital of St. Cross, Rugby.—Miss Florence Osborne, who was trained at County Tyrone Hospital, has been appointed Matron. Miss Osborne has held appointments at the Pendlebury Children's Hospital, the Sussex County Hospital, and temporarily at the London Hospital, and was senior charge nurse at the Hospital of St. Cross.

### Minor Appointments.

Chelsea Hospital.— Miss Annie Rougier has been appointed Charge Nurse at the Chelsea Infirmary in place of Miss Louisa Scott, who has been appointed Superintendent Nurse of the infirmary wards at the Maidenhead Workhouse. Miss Rougier was trained at the National Hospital, Queen Square, and has had a large amount of private nursing experience. Miss Minnie Snell has been appointed Charge Nurse at this infirmary. She trained at the Grosvenor Hospital for Women and Children, where she subsequently held the post of charge nurse for several years.

Western Fever Hospital.—Miss C. A. Archibald has been appointed Assistant Matron at the Western Fever Hospital. Miss Archibald received her training at St. Bartholomew's Hospital, and for the past nine months has held the post of night superintendent at the Chelsea Infirmary. She has excellent testimonials.

Burnley Union Infirmary.—Miss Mary E. Cave has been appointed Charge Nurse of this institution. She was trained at the New Infirmary, Birmingham, and has been for three years assistant nurse at the Workhouse Infirmary, Plymouth.

### OUR CONVALESCENT FUND.

Our grateful acknowledgments are due to Miss Dunwoodie for a contribution of 2s. 6d., and to "A Reader of The Hospital" for that of £1 to our Convalescent Fund.

### For Reading to the Sick.

### WATCH!

**Verses.**

Surely the time is short,
Endless the task and art,
To brighten for the Eternal Court
A soiled earth-drudging heart;
But He, the dread Proclaimer of that hour,
Is pledged to them in Love, as to thy foes in power.
—*Keble.*

Thy cave is fixed, and zealously attends
To fill thy odorous lamp with deeds of light
And hope that reaps not shame. Therefore, be sure,
Thou, when the Bridegroom with his feastful friends
Passes to bless at the mid hour of night,
Hast gained thy entrance, Virgin wise and pure.
—*Milton.*

Unto you is given
To watch for the coming of His feet
Who is the glory of our blessed heaven.
The work and watching will be very sweet,
Even in an earthly home;
And in such an hour as you think not
He will come. —*B. M.*

Hark! what a sound, and too divine for hearing,
Stirs on the earth and trembles in the air!
Is it the thunder of the Lord's appearing?
Is it the music of His people's prayer?
Surely He cometh, and a thousand voices
Shout to the saints and to the deaf and dumb!
Surely He cometh and the earth rejoices,
Glad in His coming, Who hath sworn, "I come."
—*F. Myers.*

Be docile to thine unseen Guide,
Love Him as He loves thee;
Time and obedience are enough,
And thou a saint shalt be. —*Faber.*

**Reading.**

Work and watch, watch and work! This is the substance of the Parable of the Talents. The message comes straight from Christ's lips; it comes to us; it seems specially meant for us in these days. The master summons his servants, tells them of his intended absence, and gives them charge of the house—deputes its responsibilities upon them, so that they feel the master's absence even more influential than his presence. They were to act for him, to represent him, to conduct the affairs of the house in his name. How great the responsibility of the master's absence! Even more solemn, more urgent than his presence. Each servant is put upon his honour, his right feeling, his conscientiousness. Instead of being rendered more careless by the absence he ought to be doubly diligent and conscientious. To each is given his own separate work. As each member of the body has its own office, so has each servant of the household his separate work. As the eye cannot hear for the ear, nor the foot ply the craft of the hand; so can no servant do the work for another. There is work enough for all, and each has his own. It is for our own that we are responsible, and for no more. This should check ambition and envy and disappointment. Each servant has his own work, which no one can do for him. Let him do it well.

### Presentation.

On the occasion of Miss Harper leaving the Scarborough Hospital, where she had been matron for the last five years, she was presented by the governors, officials, and other friends of the hospital with a purse containing £100 and a framed illuminated address. The president, Mr. F. R. Giddy, in making the gift, testified to Miss Harper's high character and devotion to duty and to the very able way she had organised and managed the institution. The Rev. Canon Dolan and other members of the board also spoke in highly eulogistic terms of Miss Harper, expressing great regret at her departure and best wishes for her future welfare.

### Travel Notes.

By Our Travelling Correspondent.

### XIV.—LOURDES.

Before leaving the region of the Pyrénées I should like you to go with me in spirit to Lourdes, that wonderful spot which draws no less than 200,000 pilgrims yearly to its shrine.

### Proximity to Pau.

You will probably visit Lourdes from Pau or Canterets. From Pau it is an easy day's excursion, and may be made even by one not in robust health without undue fatigue. The journey is only three-quarters of an hour, and there is a good train at ten minutes to nine a.m., and another at noon, and there are numerous trains by which to return. This wonderful shrine visited so lavishly by nineteenth century piety, stimulated by the agonised hope of relief from all the terrible bodily ailments to which our flesh is heir, only came into conscious existence in 1858, when the little shepherd girl Bernadette Soubircus claimed to have seen the Blessed Virgin in bodily shape.

### Position of Lourdes.

The reason for the existence of the frowning fortress, of which I give you a sketch, is that Lourdes is both very near the Spanish frontier, and also stands on an eminence which commands the four roads leading respectively to Tarbes, Bagnères, Argelès, and Pau. This castle, now almost overlooked in the overpowering claims of the grotto to notoriety, is of considerable interest. It is entered by a drawbridge and a door only 4 feet in height and about 20 inches wide, so jealously was it guarded in ancient times. The village, now swollen to the dimensions of a town, is most beautifully situated, and before it was ruined by the greedy swarm of hotel keepers, dealers in objets de piété, and the like, must have been a calm, peaceful, and most attractive spot.

Lourdes, with the Pilgrimage Church to the Left.

### The National Pilgrimage.

This takes place in August, and for those in perfect health, it is quite worth while to spend two or three days at Lourdes to witness this extraordinary demonstration. I have done so myself, and would not have missed so remarkable and, I must add, painful sight on any account.

### Monsieur Zola's Book.

Most people probably read this realistic but most truthful account of the national pilgrimage, so that it is needless for me to enter on the subject; but I think it must naturally strike those who have not been spectators as grossly exaggerated. This is not the case—Monsieur Zola has exaggerated nothing of the grievous sights witnessed. It is, indeed, more pitiful than words can describe.

### The Grotto.

The Massavielle Grotto, whence flows the miraculously healing water, is small in size, with a projection of rock within on which is placed a statue of the Blessed Virgin in the very spot where Bernadette asserted that she saw her in 1858. This rock is black and polished with the kisses of the faithful, and is perpetually surrounded with burning tapers, the rough walls and overhanging rocky roof are profusely hung with crutches and splints and surgical appliances of all kinds, no longer needed by those who professed to have been entirely healed by the Madonna's intervention.

### The Fountain and Baths.

The water is led from its source behind the Grotto to a series of baths by means of a long pipe fitted with taps, at which you can drink and also fill any cases or bottles that are brought by the faithful for transportation to other localities, and sufferers at a distance. The baths, which are very cold, are close by, and outside you may almost always see friends and relatives of invalids kneeling with outstretched arms, beseeching the clemency of the Blessed Virgin.

### The Basilica.

This is one of the most remarkable churches in the whole world—by no means beautiful, but unique; the entire walls are covered with votive offerings of all sorts and kinds from believers of every country, swords offered up in gratitude that the owners had returned in safety from war, thousands of gold and silver hearts, which round the Triforium are used to spell the words addressed to Bernadette; then under the Basilica is the crypt cut in the solid rock with a large number of confessionals and the walls entirely decorated with grateful and most pathetic inscriptions in gilt letters upon small marble slabs, such as "P. D., three years old, has been preserved to the love of his parents"—"I was crippled in both legs, and now I am well," and an artless prayer, "May her protection extend to the glass trade," and many others equally strange and frank. Seated in front of the Basilica is a priest always in attendance to bless the articles bought in the town—rosaries, chaplets, medals, &c. At the time of the national pilgrimage there is a torchlight procession, which goes all round behind the Basilica and Calvary, and however little one may be in sympathy with the religious fanaticism that prompts some of the extravagances observed at the time, one cannot but be deeply moved by the despairing agony that prompts so many hundreds to endure untold suffering and misery in the blessed hope of purchasing health for themselves or those they love more dearly than themselves.

### The Old Town.

This is now fairly swamped by the ugly eruption of scores of hotels, pensions, hospitals, and shops, but patient research will discover the birthplace of Bernadette Soubirous in the Rue des Petits-Fossés. It is, or was a very short time since, in a state of entire neglect, but one looks with strange interest at that mere hovel whence Bernadette started on that eventful February morning. I must say that the recollections left by a visit to Lourdes are sad ones—must necessarily be so, I think, to anyone of a reflecting mind. Everything is so considered from a commercial point of view; the only comfort is to see the whole-hearted and unselfish devotion of the sisters of charity who accompany and minister to the many loathsome cases brought down in the national pilgrimage, more especially in that terrible "white train" depicted with such fearful truthfulness by M. Zola.

### HINTS TO TRAVELLERS.

Do not burden yourselves with numerous hand packages. Two is as much as one person can manage with ease—a dressing bag and tea basket, whilst your companion can have her dressing bag and the rugs. Do not allow anything to be too cumbersome for the racks or too high to go under the seat. Remember that Continental trains are always full; our neighbours do not believe in seats to spare, and the luxury of unoccupied space for which no one pays.

### A Few Remarks as to Clothes.

Do not take too many—it is the usual fault. If you are not going into much gaiety, two good tailor gowns (coats and skirts), with a variety of waistcoats and blouses, and one good plain evening dress for theatres or an occasional festivity is all that is needed. Much dress at table d'hôte is bad form. Drill dresses are pleasant in hot weather, but it is wise to bear in mind that washing is expensive abroad. Above all things, have comfortable boots and shoes. Avoid patent leather, very few feet will stand a long walk in that covering. Whether you are robust or delicate always wear woollen underclothing, and take flannel or merino night-dresses as a precaution against damp beds.

### TRAVEL NOTES AND QUERIES.

Rules in regard to Correspondence for this Section.—All questioners must use a pseudonym for publication, but the communication must also bear the writer's own name and address as well, which will be regarded as confidential. All such communications to be addressed "Travel Editor, 'Nursing Mirror,' 28, Southampton Street, Strand." No charge will be made for inserting and answering questions in the inquiry column, and all will be answered in rotation as space permits. If an answer by letter is required, a stamped and addressed envelope must be enclosed, together with 2s. 6d., which fee will be devoted to the objects of the "Hospital Convalescent Fund." Any inquiries reaching the office after Monday cannot be answered in "The Mirror" of the current week.

Madeira (Meteor).—I fear in no way can it be done cheaply; the British and African line, which goes weekly from Liverpool, is the most reasonable, £10 first-class; all the other lines are £15, but I am told the B. and A. is very satisfactory.

Riviera (Lunatic).—I admire your pluck and wish you all success. As you are near a garrison town, and not easily daunted, I should beg the loan of the military ambulance as far as Basingstoke; from London help is easily obtained by payment from the hospitals, and the patient must make the journey straight through. Fortunately, money need not be considered, which simplifies matters considerably; the cost of two trained bearers and loan of stretcher would not be very great, and prove an immense boon.

Castellamere (Lily).—I think you would find it unduly hot in the summer; it would be better to leave early in June and go up to the mountains.

Alassio (Three cornered).—We published a short account of a good nursing home there in the issue of January 14th. You will find all the information you need in it.

### Pension Fund Nurses.

### MISS BURNS' WEDDING GIFT.

We have to acknowledge the receipt of contributions from Nurse E. Hoskier and from Policy No. 4,812. Nurses J. Sewell and Mary Ann Smith are reminded that no smaller contribution than 6d. can be accepted for this object; if they will send a stamped addressed envelope their contribution shall be returned.

### Wants and Workers.

The Matron, Dorset County Home for Nurses, Dorchester, wishes to hear of an elderly Lady or Nurse who, having given up nursing, would be glad of a home. Coals, light, and £10 yearly would be given in return for keeping a small house, with no other occupants, clean and tidy, and forwarding any letters and telegrams to another address.

### Notes and Queries.

**The contents of the Editor's Letter-box have now reached such unwieldy proportions that it has become necessary to establish a hard and fast rule regarding Answers to Correspondents. In future, all questions requiring replies will continue to be answered in this column without any fee. If an answer is required by letter, a fee of half-a-crown must be enclosed with the note containing the enquiry. We are always pleased to help our numerous correspondents to the fullest extent, and we can trust them to sympathise in the overwhelming amount of writing which makes the new rules a necessity.**

**Every communication must be accompanied by the writer's name and address, otherwise it will receive no attention.**

*Curative Gymnastics.*

(245) Can you tell me whether gymnastic exercises would be beneficial to an adult of 30 who has always had slight lateral curvature of the spine, and a slight stoop? If gymnastics are likely to do her good, will you give the address of any gymnastic class for women in London? —*Straightback.*

Much good may be done by gymnastics in some cases, even at a more advanced age, but they must be undertaken under the supervision of a medical man. A mistaken exercise may be harmful. 2. We cannot recommend special institutions.

*Hysteria.*

(246) What is the cause of hysteria? 2. Is there any treatment for it? 3. Would it deter any one entering a situation?—*Companion.*

Can you tell me of any home or hospital that would take a case of pure hysteria, where the patient could have proper treatment with a view to cure? The age is 28, and the hysteria came on after severe mental shock. She is a lady in poor circumstances, who ought to be earning her living. Her friends could make a small weekly payment, but cannot afford much.—*Nell.*

1. The causes are various, and some of them obscure. 2. Hysteria generally yields to medical treatment, especially when the patient loyally co-operates in her cure. 3. Would anyone knowingly expose themselves to the trouble and annoyance of dealing with an hysterical employée? We fancy not. "Nell" might write to the special hospitals for nervous diseases.

*Home for Consumptives.*

(247) Could you kindly let me have particulars (or address where I could obtain the same) of any home or hospital for a gentleman in the first stage of phthisis? He does not wish to go too far from England. —*Margaret.*

Your patient should consult a specialist on the subject.

*Sterilising Milk.*

(248) I shall be greatly obliged if you will tell me how I can best sterilise milk for family use in the most simple and labour-saving way, so that the milk shall be made quite safe and not likely to catch while heating, and not requiring elaborate apparatus difficult to clean and keep in order?—*Mother W.*

Just before milk boils it will be seen to heave and seethe in the pan. When this takes place it will be practically sterilised, and should be poured off into a clean jug at once. Therefore a clean pan—a brass one preferably, because the thickness prevents the milk burning—is the only apparatus necessary to successfully sterilise milk. But the milk must be watched while boiling or it will boil over. Milk can, however, be Pasteurised very easily by using Aymard's apparatus, which is very simple and easy to manage.

*Vacant Matronships.*

(249) Will you kindly inform me how and where I could hear of any possible matronship in connection with some of the smaller sanatoriums for consumptives, which are apparently being started in England? —*E. R.*

Scan the advertisements appearing in our columns and those in the poor-law journals, and apply in the usual way.

*Institutions.*

(250) Although I have been appointed to a vacancy as probationer I am undecided whether to become a nurse or not. Will you tell me if there are any nursing institutions worked on the co-operative system in Leeds, Scarborough, or York? and if not, are there any institutions in Leeds which pay a definite salary to the nurses on its staff?—*Anxious.*

At the root of all good nursing lies a genuine love of the work. Indecision, after obtaining an appointment as probationer, does not promise this essential qualification. We have no information of institutions worked on the co operative system in any of the towns named.

*Phthisis.*

(251) Would you kindly say whether there is any hospital in England where a nurse who is in the early stage of phthisis could be treated free. —*Sister M.*

The Hospital for Consumption and Diseases of the Chest, Brompton, S.W. (admission by subscriber's letter), the City of London Hospital for Diseases of the Chest, Victoria Park (by governor's recommendation), and the North London Hospital for Consumption, Mount Vernon, Hampstead. N.W. (by a governor's letter). Write to the secretaries of each and ask for directions how to obtain the letters.

*Outdoor Uniforms.*

(252) I have strong objections to wearing "outdoor" nurse's uniform. Will you inform me whether a hospital committee or matron can compel me to wear it, and under what conditions?—*Liberty.*

The question is not one of compulsion, but of right feeling and discipline. A committee or matron might feel justified in dismissing a nurse who needed to be compelled to obey their instructions. On the other hand such arrangements ought to be specified at the time of engagement. The wearing of outdoor uniform is often a matter of great convenience, but in our opinion, except perhaps in the case of young probationers, it ought not to be compulsory.

## Figures and Tables

**Figure f1:**
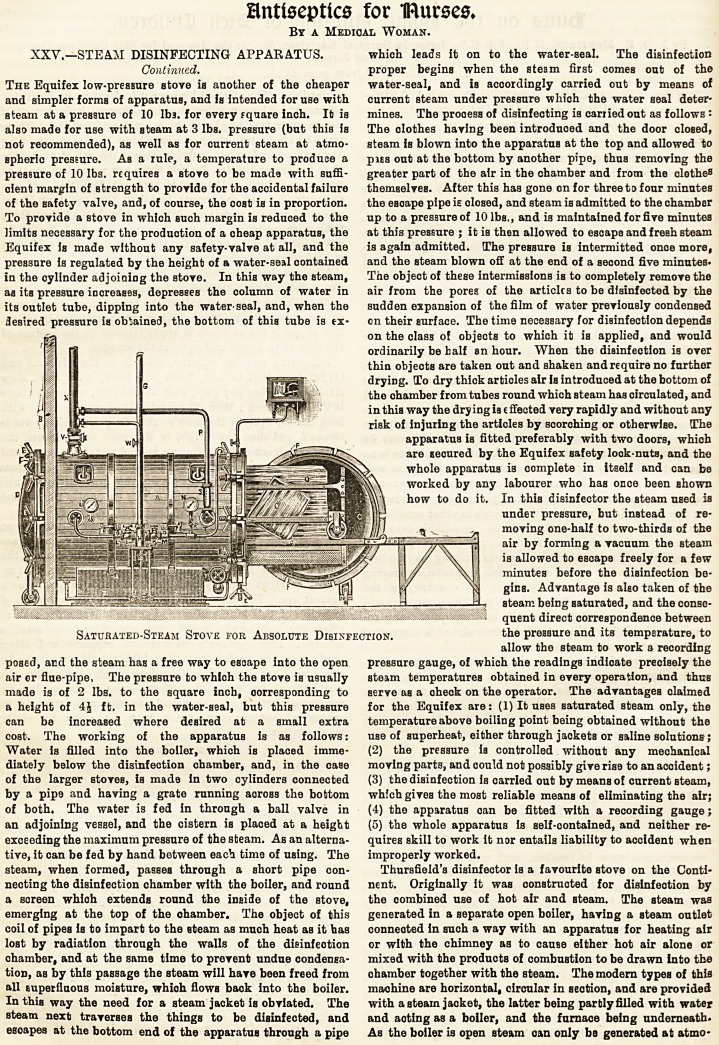


**Figure f2:**